# Reproducibility of CMR right ventricle volumetric measurements is independent of reader experience under a standardized protocol

**DOI:** 10.1186/1532-429X-16-S1-P272

**Published:** 2014-01-16

**Authors:** Murilo Foppa, Arman Ashrafi, Carol J Salton, Michael L Chuang, Warren J Manning

**Affiliations:** 1 Cardiology, Beth Israel Deaconess Medical Center, Boston, Massachusetts, USA; 2 NHLBI's Framingham Heart Study, Framingham, Massachusetts, USA

## Background

The right ventricle's complex geometry makes it challenging to measure its size and function. Though volumetric CMR is generally considered the gold standard, standardized reading protocols are necessary. We sought to investigate whether a specific reading protocol can supersede cardiology training in right ventricular (RV) measurements. We aimed to evaluate the reproducibility of RV measurements done by both an experienced and a novice CMR reader.

## Methods

During a training period an experienced (Level III trained) CMR reader and a CMR novice (high school student) jointly interpreted 50 cine SSFP studies obtained in the left ventricular short-axis orientation as contiguous stack encompassing both ventricles in their entirety. The standardized endocardial tracing protocol was delimited by the pulmonary valve and the tricuspid valve planes; interpretation was augmented using cine-review and cross-referencing with orthogonal cine images. RV stroke volume (RVSV) and ejection fraction (RVEF) were calculated from end diastolic volume (RVEDV) and end systolic volume (RVESV). After the training period, we randomly selected 24 anonymized datasets from a representative sample (50% men; median age 63 years[range 48-78]), and the two readers independently read the studies twice, in a random order and on different days. Reproducibility was evaluated with coefficients of variation (CV) and concordance correlation coefficients (Rho).

## Results

Mean ± SD measurements and CV did not differ between the experienced and the novice reader, respectively, for RVEDV (124.4 ± 30.1 vs 125.0 ± 30.1 mL; CV: 0.05 vs 0.04; P = NS), RVESV (45.6 ± 14.4 45.8 ± 13.4 mL; CV: 0.11 vs 0.09; P = NS), RVSV (78.9 ± 18.8 vs 79.2 ± 18.7 mL; CV: 0.05 vs 0.03; P = NS), RVEF (63.6 ± 5.4 vs 63.4 ± 5%; CV: 0.04 vs 0.04: P = NS). The concordance correlation coefficients and 95% limits of agreement are shown in the Table 1.

**Table 1:** Intrarreader and interreader concordance correlation coefficients (Rho) and mean differences with 95% limits of agreement

	Experienced Reader		Novice reader		Interreader	
	**Rho(95%CI)**	**Diff (95%CI)**	**Rho(95%CI)**	**Diff (95%CI)**	**Rho(95%CI)**	**Diff (95%CI)**

RVEDV	0.98(0.96-1.0)	1.1 (-11,13)	0.99(0.98-1.0)	1.35 (-8,10)	0.94(0.90-0.98)	0.1(-9,9)

RVESV	0.91(0.85-0.97)	3(-7,13)	0.96(0.92-0.98)	1.8 (-6,10)	0.93(0.89-0.98)	0.1(-9,9)

RVSV	0.97(0.94-0.99)	-1.8(-11,7)	0.99(0.99-1.0)	-0.5(-5,4)	0.97(0.96-1.0)	-0.7(-8,7)

RVEF	0.81(0.68-0.95)	-1.8(-7,4)	0.92(0.88-0.98)	-0.9(-5,3)	0.89(0.80-0.98)	-0.2(-5,4)

## Conclusions

All global volumetric CMR RV measurements were highly reproducible, suggesting that a standardized reading protocol overcomes the complex RV geometry. Background CMR level of training did not affect these results. The high reproducibility and feasibility of applying a specific training protocol may impact research, allowing smaller sample sizes and better logistics for reading as well as for serial clinical assessment.

## Funding

Dr Murilo Foppa has received support from CAPES-Brazil (9601-11-2).

